# Endoplasmic reticulum stress induces secretion of high-mobility group proteins and is associated with tumor-infiltrating lymphocytes in triple-negative breast cancer

**DOI:** 10.18632/oncotarget.11010

**Published:** 2016-08-02

**Authors:** In Ah Park, Sun-Hee Heo, In Hye Song, Young-Ae Kim, Hye Seon Park, Won Seon Bang, Suk Young Park, Jeong-Hyon Jo, Hee Jin Lee, Gyungyub Gong

**Affiliations:** ^1^ Department of Pathology, University of Ulsan College of Medicine, Asan Medical Center, Seoul, Korea; ^2^ Asan Center for Cancer Genome Discovery, Asan Institute for Life Sciences, University of Ulsan College of Medicine, Asan Medical Center, Seoul, Korea; ^3^ Department of Pathology, Gangnam Severance Hospital, Yonsei University College of Medicine, Seoul, Korea

**Keywords:** breast carcinoma, tumor-infiltrating lymphocytes, endoplasmic reticulum, high-mobility group protein

## Abstract

**Background:**

Although the prognostic and predictive significance of tumor-infiltrating lymphocytes (TILs) in triple-negative breast cancer (TNBC) have been shown, the cause of the TIL influx is unclear. Here, we investigated whether extracellular secretion of HMGN1 is associated with TIL influx, as well as increased endoplasmic reticulum stress (ERS), in human TNBC.

**Methods:**

We reviewed the slides of 767 patients with TNBC and evaluated the TIL levels. We also assessed the expression of HMGs and several ERS-associated molecules using immunohistochemical staining. Western blot analysis of human TNBC cell lines and pharmacological ERS inducers was used to determine if HMGN1 migrates from the nucleus to the extracellular space in response to ERS.

**Results:**

On immunohistochemical staining, either higher nuclear or cytoplasmic expression of both HMGB1 and HMGN1 was significantly associated with ERS. TILs showed a positive correlation with the cytoplasmic expression of the HMGs. Western blot analysis of TNBC cell lines showed that ERS induction resulted in the secretion of HMG proteins.

**Conclusions:**

This is the first study to elucidate the associations among ERS, secretion of HMGs, and degree of TILs in TNBCs. Understanding the mechanisms of TIL influx will help in the development of effective immunotherapeutic agents for TNBC.

## INTRODUCTION

The strong prognostic and predictive significance of tumor-infiltrating lymphocytes (TILs) in triple-negative breast cancer (TNBC) have been documented [1–7]. However, the mechanisms of TIL influx in breast cancer remain to be elucidated.

Preclinical studies have shown that radiotherapy and various chemotherapeutic agents, such as anthracycline, taxane, and cyclophosphamide, can induce anticancer immune responses called immunogenic cell death [8, 9]. This process activates the immune system and consequently augments the anti-tumor effects of chemotherapy and radiotherapy. Immunogenic cell death is mainly mediated by damage-associated molecular patterns (DAMPs), including calreticulin, ATP, and high-mobility group (HMG) box 1 (HMGB1) [8, 10]. HMG proteins are abundant and universal nucleosome-binding proteins that are subclassified into three groups by their structural motifs: HMGA, HMGB, and HMGN [11]. HMGB1 is well known as a DAMP secreted from injured or dying cells that activates the innate immune system by binding to receptor for advanced glycation end products (RAGE) [12, 13] or toll-like receptors (TLRs) [14]. Binding of HMGB1 to TLR4, following the calreticulin exposure on the surface of tumor cells and ATP secretion, is associated with the recruitment and maturation of dendritic cells (DCs) and the consequent proliferation of tumor-reactive CD8+ T lymphocytes, which are presented tumor-associated antigen by mature DCs [10].

The combined action of reactive oxygen species and endoplasmic reticulum (ER) stress (ERS) is another key component of immunogenic cell death because they activate danger signalling pathways [8]. In eukaryotic cells, the ER is responsible for organelle calcium storage, lipid biosynthesis, and protein folding and trafficking [15]. Physiological and pathologic conditions that disrupt ER protein folding processes and lead to the accumulation of misfolded proteins in the ER are called ERS processes. To maintain the homeostasis of the cell, ERS activates the unfolded protein response (UPR), which is initiated by liberating binding protein (BiP) from the three ERS sensors, PERK, IRE1, and ATF6. Moreover, various downstream signalling pathways are activated upon UPR initiation [16]. In malignancy, the cells experience ERS through various events, including increased protein synthesis, hypoxia, and nutrient depletion. Therefore, it is presumed that increased ERS in cancer cells causes DAMP release, even before the use of anticancer therapeutics.

We recently showed that high cytoplasmic expression of HMGB1 and HMGN1 was correlated with larger amounts of TILs in HER2-positive breast cancers [17]. We also recently found that high cytoplasmic expression of HMGB1 was significantly associated with abundant TILs and high numbers of CD8+ cells in two cohorts of patients with TNBC [18]. However, the relationship among ERS, HMG release, and TIL influx in the tumor microenvironment remains to be evaluated.

In our present study, we evaluated the relationship among the amount of TILs and expression of HMG proteins and ERS molecules in TNBC tissue samples from a TNBC patient cohort. The hypothesis that HMGs were released upon ERS was also explored using western blot analysis of primary TNBC cell lines.

## RESULTS

### Expression of ERS molecules and HMGs in TNBC

The protein expression levels were dichotomized by their mean value, with immunohistochemical staining used to determine the percentage of tumors with a higher expression level of HMG (HMGB1 and HMGN1) in both the nucleus and cytoplasm and of several ER stress-associated molecules (PERK, p-eIF2a, and XBP-1) in the cytoplasm. High nuclear expression of HMGB1 and HMGN1 was identified in 54.6% and 48.2% of TNBCs, respectively. On the other hand, high cytoplasmic expression of HMGB1, HMGN1, PERK, p-eIF2a, and XBP-1 was identified in 43.1%, 25.7%, 45.9%, 46.9%, and 41.8% of TNBCs, respectively. HMGs also showed immunopositivity in normal epithelial cells of terminal duct lobular units in some cases.

### Characteristics of tumors with high ERS molecules

Using the ERS-associated molecules, the patient group was divided into three subgroups according to the dichotomized p-eIF2a and XBP-1 expression levels. PERK was not included because it shares the same signalling pathway as p-eIF2a, which is downstream of PERK. The subgroup with low ERS was defined as having low cytoplasmic expression of both p-eIF2a and XBP-1 (253 cases, 34.0%), whereas the patients with high expression of one of these two molecules were designated as having intermediate ERS (331 cases, 44.5%). The subgroup showing high expression of both p-eIF2a and XBP-1 was considered to have high ERS (160 cases, 21.5%). Variable clinicopathologic parameters, including the expression pattern of HMG proteins and the other ERS-associated proteins in TNBC, were analyzed (Table 1). Higher histologic grade (*p*<0.001), large amount of TILs (*p*<0.001), and higher expression of cytoplasmic PERK (*p*<0.001) were associated with high ERS. High cytoplasmic expression of HMGN1 (*p*=0.001) and both nuclear (*p*=0.047) and cytoplasmic (*p*=0.036) expression of HMGB1 were also significantly associated with a high ERS.

**Table 1:** Comparison of clinicopathologic variables according to the cytoplasmic expression level of ER stress-associated proteins in triple-negative breast cancer

		Cytoplasmic p-eIF2a and XBP-1			*p*
		Low expression (*n*=253, 34.0%)	Intermediate expression (*n*=331, 44.5%)	High expression (*n*=160, 21.5%)	
Histologic grade	1&2	91 (36.0)	74 (22.4)	24 (15.0)	**<0.001**
	3	162 (64.0)	257 (77.6)	136 (85.0)	
pT	1	109 (43.1)	131 (39.6)	78 (48.8)	0.375
	2	134 (53.0)	189 (57.1)	76 (47.5)	
	3	9 (3.6)	11 (3.3)	6 (3.8)	
	4	1 (0.4)	0 (0.0)	0 (0.0)	
LN metastasis	Negative	156 (61.7)	220 (66.5)	108 (67.5)	0.372
	Positive	97 (38.3)	111 (33.5)	52 (32.5)	
TIL	<10%	83 (32.8)	69 (20.8)	25 (15.6)	**<0.001**
	20%–30%	62 (24.5)	76 (23.0)	29 (18.1)	
	40%–60%	41 (16.2)	74 (22.4)	42 (26.3)	
	>60%	67 (26.5)	112 (33.8)	64 (40.0)	
Cytoplasmic PERK	Low	170 (67.2)	165 (49.8)	78 (48.8)	**<0.001**
	High	83 (32.8)	166 (50.2)	82 (51.3)	
Nuclear HMGB1	Low	117 (50.0)	147 (45.9)	60 (37.5)	**0.047**
	High	117 (50.0)	173 (54.1)	100 (62.5)	
Cytoplasmic HMGB1	Low	148 (63.5)	174 (54.4)	83 (51.9)	**0.036**
	High	85 (36.5)	146 (45.6)	77 (48.1)	
Nuclear HMGN1	Low	139 (55.4)	164 (49.7)	76 (47.5)	0.230
	High	112 (44.6)	166 (50.3)	84 (52.5)	
Cytoplasmic HMGN1	Low	204 (81.3)	239 (72.4)	103 (64.4)	**0.001**
	High	47 (18.7)	91 (27.6)	57 (35.6)	

Low expression, low p-eIF2a and low XBP-1 expression; intermediate expression, low p-eIF2a and high XBP-1 expression, or high p-eIF2a and low XBP-1 expression; high expression, high p-eIF2a and high XBP-1 expression

### TILs and expression of HMG proteins and ERS molecules in TNBC

We analyzed the correlation among the amount of TILs and the expression levels of HMG proteins and ERS-associated molecules (Table 2). TILs showed a significant positive correlation with the cytoplasmic expression of the HMG proteins HMGB1 (rho=0.231, *p*<0.001) and HMGN1 (rho=0.147, *p*<0.001). The nuclear expression of HMGN1 was negatively correlated with TILs (rho=−0.146, *p*<0.001), whereas that of HMGB1 was not. Cytoplasmic expression of p-eIF2a and XBP-1 showed a significant positive association with TILs. PERK expression and TILs were not well correlated in our data.

**Table 2:** Correlation between the amount of TILs and the expression levels of HMG proteins and ER stress-associated molecules

	Nuclear HMGB1	Cytoplasmic HMGB1	Nuclear HMGN1	Cytoplasmic HMGN1	Cytoplasmic PERK	Cytoplasmic XBP-1	Cytoplasmic p-eIF2a
TIL	−0.014	**0.231**	−0.146	**0.147**	0.020	**0.091**	**0.153**
	(*p*=0.692)	**(*p*<0.001)**	**(*p*<0.001)**	**(*p*<0.001)**	(*p*=0.579)	**(*p*=0.011)**	**(*p*<0.001)**
Nuclear		**0.244**	**0.447**	−**0.215**	**0.267**	**0.096**	0.032
HMGB1		**(*p*<0.001)**	**(*p*<0.001)**	**(*p*<0.001)**	**(*p*<0.001)**	**(*p*=0.009)**	(*p*=0.379)
Cytoplasmic			−**0.169**	**0.288**	**0.075**	**0.176**	0.026
HMGB1			**(*p*<0.001)**	**(*p*<0.001)**	**(*p*=0.039)**	**(*p*<0.001)**	(*p*=0.479)
Nuclear				−**0.311**	**0.159**	−0.018	**0.149**
HMGN1				**(*p*<0.001)**	**(*p*<0.001)**	(*p*=0.612)	**(*p*<0.001)**
Cytoplasmic					0.022	**0.104**	**0.103**
HMGN1					(*p*=0.534)	**(*p*=0.004)**	**(*p*=0.004)**
Cytoplasmic						**0.198**	**0.216**
PERK						**(*p*<0.001)**	**(*p*<0.001)**
Cytoplasmic							**0.144**
XBP-1							**(*p***<0.001)****

### Prognostic significance of TILs and the expression of HMG proteins and ERS molecules in TNBC

The prognostic significance of TILs and other clinicopathologic variables was analyzed (Table 3). By univariate analysis, higher pathologic tumor (pT) stage, presence of lymph node metastasis, higher pTNM stage, chemotherapy regimen with both AC and taxane, lower level of TILs, and lower expression of p-eIF2a were negative prognostic factors for both disease-free and overall survival. However, there were no significant associations between the expression levels of the HMGs and patient survival. By multivariate analysis using a conditional forward stepwise algorithm, pTNM stage and TILs were independent prognostic factors for disease-free survival: pTNM stage, hazard ratio (HR) 2.281, 95% confidence interval (CI) 1.754–2.966 (*p*<0.001); TILs, HR 0.981, 95% CI 0.974–0.988 (*p*<0.001). Both of these variables were also independent prognostic factors for overall survival: pTNM stage, HR 2.030, 95% CI 1.534–2.687 (*p*<0.001); TILs, HR 0.980, 95% CI 0.972–0.988 (*p*<0.001).

**Table 3:** Univariate analyses of clinicopathologic variables and expression level of proteins affecting clinical outcomes

Variable	Disease-free survival			Overall survival		
	HR	95% CI	*p*	HR	95% CI	*p*
Age: <50 *vs* ≥50 years	0.847	0.588–1.222	0.375	1.114	0.758–1.637	0.584
Histologic grade: 1&2 *vs* 3	0.765	0.522–1.121	0.169	0.838	0.551–1.273	0.406
pT stage: 2,3&4 *vs* 1	1.998	1.356–2.945	**<0.001**	1.752	1.161–2.642	**0.008**
Lymph node metastasis: positive *vs* negative	2.557	1.794–3.645	**<0.001**	2.309	1.576–3.384	**<0.001**
pTNM stage: 2&3 *vs* 1	2.305	1.477–3.600	**<0.001**	1.938	1.221–3.076	0.005
Radiation therapy: negative *vs* positive	0.816	0.543–1.225	0.327	0.770	0.500–1.187	0.770
Chemotherapy: AC *vs* ACT	0.404	0.284–0.575	**<0.001**	0.453	0.310–0.664	**<0.001**
TILs (>10% *vs* ≤10%)	0.983	0.976–0.990	**<0.001**	0.981	0.973–0.989	**<0.001**
Nuclear HMGB1 expression: high *vs* low	1.258	0.861–1.837	0.236	1.067	0.715–1.593	0.751
Cytoplasmic HMGB1 expression: high *vs* low	1.194	0.796–1.792	0.391	1.010	0.674–1.514	0.961
Nuclear HMGN1 expression: high *vs* low	1.094	0.767–1.561	0.619	0.844	0.574–1.243	0.391
Cytoplasmic HMGN1 expression: high *vs* low	0.920	0.610–1.390	0.694	1.115	0.730–1.703	0.615
PERK expression: high *vs* low	0.882	0.616–1.262	0.492	0.726	0.489–1.077	0.112
p-eIF2a expression: high *vs* low	0.646	0.447–0.993	**0.020**	0.635	0.426–0.946	**0.025**
Cytoplasmic XBP-1 expression: high *vs* low	0.993	0.690–1.427	0.968	0.934	0.631–1.382	0.732

HR, hazard ratio; CI, confidence interval; AC, anthracycline and cyclophosphamide; ACT, anthracycline, cyclophosphamide, and taxane; TIL, tumor-infiltrating lymphocyte

### Release of HMG proteins upon ERS

**Figure 1:**
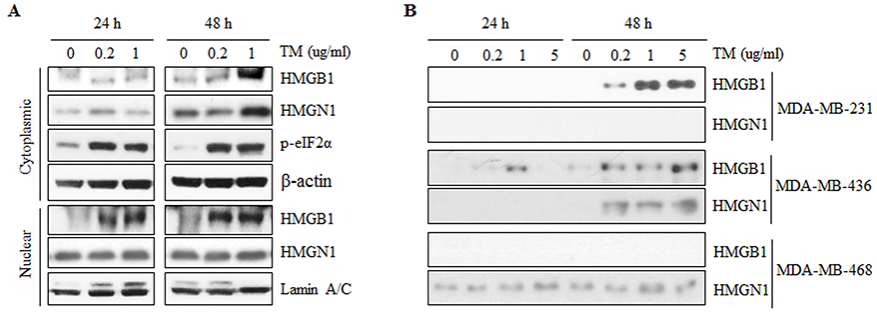
Expression and secretion of HMGB1 and HMGN1 during tunicamycin-induced ER stress in triple-negative breast cancer cell lines **A.** ER stress was induced by treatment with tunicamycin (TM) at the indicated concentrations for 24 or 48 h. After TM treatment for 24 or 48 h, cytoplasmic and nuclear proteins were extracted from MDA-MB-468 and separated on SDS-PAGE gels. p-eIF2α was detected for a indicator of ER stress induction. Protein expression of HMGB1 and HMGN1 was confirmed in cytoplasm and nuclear fractions. **B.** Secretion of HMGB1 and HMGB1 were assessed in conditioned media which collected after treatment with tunicamycin for 24 and 48 hours in MDA-MB-23, -436 and -468 by western blotting.

The expression levels of HMGB1, HMGN1 and p-eIF2α were assessed in MDA-MB-468, a TNBC cell line, during tunicamycin-induced ERS by western blotting (Figure 1A). Tunicamycin is a widely used ERS inducer that can also induce autophagy in breast cancer cell lines [19]. An increased level of p-eIF2α, one of the most well-known ERS-associated molecules, was observed after treatment with 0.2 or 1.0 μg/ml of tunicamycin for 24 h or 48 h (Figure 1A).

The cytoplasmic expression of HMGN1 was increased at 48 hours in a dose-dependent manner, whereas the nuclear expression of HMGN1 was not significantly changed after tunicamycin treatment (Figure 1A).

To confirm the association between ERS and release of HMGB1 and HMGN1 to extracellular space, secretion of HMGB1 and HMGN1 into culture media was assessed in three TNBC cell lines including MDA-MB-231, MDA-MB-436, and MDA-MB-468 (Figure 1B). HMGB1 was detected in conditioned media from MDA-MB-231 by tunicamycin treatment for 48 hours while HMGN1 was not observed (Figure 1B). In addition, HMGB1 and HMGN1 were detected during tunicamycin-induced ERS in conditioned media from MDA-MB-436, whereas HMGN1 was observed in secretome of MDA-MB-468 and the expression level was increased after tunicamycin treatment (Figure 1B). These results support our hypothesis that ERS would induce the secretion of HMGB1 and HMGN1 into the extracellular space.

## DISCUSSION

To the best of our knowledge, our present study is the first to elucidate the associations among ERS, HMG secretion, and the degree of TILs in TNBCs. We observed high nuclear expression of HMGB1 and HMGN1 in about half of our TNBC patients (54.6% and 48.2%, respectively) whereas high cytoplasmic expression of HMGB1 and HMGN1 was identified in 43.1% and 25.7% of these patients, respectively. After subdivision of the patients into three subgroups using dichotomized p-eIF2a and XBP-1 expression, patients with higher ERS represented 21.5% of all patients (160 of 767 patients). The ERS-associated transcription factor XBP-1 is known to be specifically activated in TNBC and not in other subtypes of breast cancers [20], but the degree of expression of other UPR regulators in each subtype is unknown.

Recent evidence shows that responses to ERS are new and distinct molecular signatures in TNBC [20, 21]. Further experimental studies have found that ERS induction, in turn, induced autophagy and apoptosis in breast cancer cell lines under the regulation of the Akt/mTOR pathway [22] and IRE1/JNK/beclin-1 [19], with the authors suggesting that ERS promotion in breast cancer may be a therapeutic target of TNBC.

Zhu *et al* [23] reported that HMGB1 played important roles in ERS as well as the maturation and activation of mouse splenic DCs. HMGN1 is also an endogenous mediator that promotes the recruitment and activation of DCs, with Hmgn1^−/−^ mice showing both deficient DC recruitment and decreased production of inflammatory cytokines [24]. However, HMGN1 had not previously been studied with ERS inducers or its associated UPR sensors, so our study is the first to elucidate the association between ERS and the extracellular secretion of HMGN1.

Recently, we observed that high cytoplasmic expression of HMGB1 was significantly associated with larger amounts of TILs and high numbers of CD8+ cells in tissue samples from patients with TNBC [18]. In addition, high cytoplasmic expression of not only HMGB1, but also HMGN1 was well correlated with the amount of TILs in our present study. As DCs are known to approach and make contact with dying cancer cells and to take tumor-associated antigens and cross-present them to cytotoxic T cells [10, 25], further functional studies using a microfluidic device to show the real-time influx of TILs following the migration of HMGs and DCs are warranted. These are in the planning phase in our laboratory.

On the contrary to HMGN1, it seemed that cytoplasmic expression of HMGB1 was not always followed by release of the protein on Western blot assay (Figure 1). This discordance may varies by cell lines which express The ERS-associated molecules in different patterns (Supplementary Figure S3). Moreover, secretion of HMGB1 has been reported to be regulated by other factors such as caspase 1[26] or acetylation of lysine residues by SIRT-1[27]. These variables might account for the discordant experimental result, which should be confirmed by controlled further studies. In terms of patient survival, only the cytoplasmic expression of p-eIF2a showed significant prognostic values for both disease-free and overall survival by univariate analysis. However, neither remained as prognostic factors in multivariate analysis, which was assumed to be because they have significant positive correlation with the amount of TIL influx. The reason why HMGs did not show prognostic value in TNBC can be explained by various roles of HMGs. HMGB1 has been known to have important roles not only as DAMP, but also in cancer cell migration, metastasis, angiogenesis and tumor growth [28–31]. These tumorigenic activities might offset the positive correlation of HMGs with TILs and consequent impact on prognosis.

In summary, we have observed that a high cytoplasmic expression of HMGs is significantly positively correlated with larger amounts of TILs in TNBC patients. Cytoplasmic expression of the ERS molecules XBP-1 and p-eIF2a was also found to be positively correlated with the amount of TILs. Western blot analysis revealed increased cytosolic expression and secretion of HMGN1 into the medium of TNBC cell lines treated with the ERS inducer tunicamycin. Thus, we conclude that it is reasonable to assume that HMGs are secreted from TNBC cells to the extracellular space under ERS conditions and are associated with the influx of TILs into the tumor stroma. Further efforts to understand the mechanisms of TIL influx in the context of ERS and HMGs might be of considerable help in the development of effective immunotherapeutic agents for the treatment of TNBC.

## MATERIALS AND METHODS

### Patients and tissue specimens

The present study included 767 female TNBC patients who underwent surgery for primary breast cancer between 2004 and 2010 at Asan Medical Center, Seoul, Korea. All patients were preoperatively chemo- and radiotherapy naïve and underwent adjuvant treatment. The adjuvant chemotherapy regimen, standard of selection for the study, and other characteristics of the patients were the same as described in our previous study [32]. Clinicopathologic information was obtained from the medical records and surgical pathologic reports. Exemption from informed consent after de-identification of information was approved by the Institutional Review Board of Asan Medical Center.

### Histological evaluation

The haematoxylin and eosin-stained slides were reviewed by two pathologists (H.J.L. and G.G.). Slides were histopathologically analyzed for TILs (defined as the percentage of stroma of invasive carcinoma infiltrated by lymphocytes in 10% increments; if less than 10% of the stroma was infiltrated by TILs, 1% or 5% criteria were used; all available full-sections were evaluated) [1, 33], histologic subtype and grade, tumor size, pT stage, pN stage, and lymphovascular invasion. The histologic type was defined based on the 2012 WHO classification criteria, and the histologic grade was assessed using the modified Bloom–Richardson classification [34].

### Tissue microarray construction and immunohistochemical evaluation

Formalin-fixed paraffin-embedded tissue samples were arrayed with a tissue-arraying instrument as previously described [35]. Each sample was arrayed in three 1-mm diameter cores to minimize tissue loss and overcome tumor heterogeneity. Tissue microarray sections were stained with an automatic immunohistochemical staining device (Benchmark XT; Ventana Medical Systems, Tucson, AZ). Antibodies to HMGB1 (1:200; Cat No. ab18256; Abcam, Cambridge, UK), HMGN1 (1:1000; Cat No. ab5212; Abcam), PERK (1:200; Cell Signaling Technology, Danvers, MA), p-eIF2α (1:200; Cat No. ab32157; Abcam), and XBP-1 (1:75; Cat No. ab37152; Abcam) were used. Protein expression was evaluated as a four-value intensity score (0, 1, 2, and 3). (Supplementary Figure S1 and S2) The percentage of nuclear and/or cytoplasmic expression was also evaluated. An ‘immunoreactive score’ was generated as the product of the intensity and the percentage of positive cells. The immunoreactive scores were dichotomized by the mean value of the expression of each protein.

### Cell culture and ERS induction

MDA-MB231, MDA-MB-436, and MDA-MB-468 cell lines were cultured in Dulbecco's Modified Eagle Medium (Cat No. 11995, Life Technologies, Grand Island, NY) containing 10% fetal bovine serum (Cat No. 16000; Invitrogen) and 1% penicillin/streptomycin (Cat No. 15140; Invitrogen) in the presence of 5% CO_2_ at 37°C. To induce ER stress, cells were treated with 0, 0.2, 1.0, or 5.0 μg/ml tunicamycin (TM, Cat No. T7765; Sigma-Aldrich) for 24 or 48 hours in DMEM containing 2% fetal bovine serum.

### Protein isolation and western blotting

A subcellular fractionation protocol provided by Thermo Scientific (Cat No. 78835; Waltham; MA) was used to isolate cytosolic and nuclear protein from cells. The protein concentrations of the cell lysates were measured using the Pierce BCA Protein Assay Reagent Kit (Cat No. 23225). Conditioned medium was collected from the supernatant by centrifugation for 5 min at 1500 rpm. Ten micrograms of proteins were separated by 15% SDS-PAGE. After electrophoresis, proteins were transferred to a polyvinylidene difluoride membrane (Millipore, Bedford, MA). The membrane was incubated with anti-HMGB1 antibody (Cat No. ab18256; Abcam), anti-HMGN1 antibody (Cat No. ab5212; Abcam), anti-p-eIF2α antibody (Cat No. ab32157; Abcam), anti-lamin A+C antibody (Cat No. ab108595; Abcam), or anti-β-actin antibody (Cat No. ab8227; Abcam) overnight at 4°C, followed by incubation with secondary antibodies for 1 h at room temperature. The Promega Western Blot Detection System (Cat No. W1008; Madison; WI) was used to detect immunoreactive proteins.

### Statistical analysis

All statistical analyses were performed using SPSS statistical software (version 18; SPSS, Chicago, IL). A chi-square test, linear-by-linear association test, Spearman's correlation, log-rank test, and Cox proportional hazards regression model were used as appropriate. All tests were two-sided and statistical significance was set at 5%.

## SUPPLEMENTARY METHODS AND FIGURES


