# Supplementary material for: Endoplasmic reticulum stress induces secretion of high-mobility group proteins and is associated with tumor-infiltrating lymphocytes in triple-negative breast cancer
Source: Oncotarget. 2016 Aug 2;7(37):59957–64. doi: 10.18632/oncotarget.11010 (PMC5312361; doi:10.18632/oncotarget.11010)
Supplement: Supplementary file 1 [file oncotarget-07-59957-s001.pdf]

Endoplasmic reticulum stress induces secretion of high-mobility group proteins and is associated with tumor-infiltrating lymphocytes in triple-negative breast cancer

SUPPLEMENTARY METHODS

Cell culture and ERS induction

Hs578t, MDA-MB231, MDA-MB-436, and MDA-MB-468cell lines were cultured in Dulbecco's Modified Eagle Medium (Cat No. 11995, Life Technologies, Grand Island, NY) containing 10% foetal bovine serum (FBS, Cat No. 16000; Invitrogen) and 1% penicillin/streptomycin (P/S, Cat No. 15140; Invitrogen) in the presence of 5% CO_2 at 37°C. BT 20 was cultured in RPMI 1640 (Cat No. A10491-01, GIBCO) with 10% FBS and 1% P/S.

Extraction of RNA and polymerase chain reaction (PCR)

Total cellular RNA was isolated using the TRIzol Reagent (Invitrogen) following the manufacturer's instructions. Briefly, single-stranded cDNA synthesis was performed with 2 μg total RNA using an oligo(dT)18primer and the Omniscript Reverse Transcriptase(Qiagen, Germany). Gene-specific primers for HMGB1, HMGN1 and GAPDH were designed using Primer 3, a web-based primer design tool. The mRNA levels of HMGB1, HMGN1 and GAPDH were determined by PCR using the following primers: HMGN1sense 5'-AAG GAA GAG CCC AAG AGG AG-3', HMGN1 antisense 5'-AGTCTTCGTTTCCCCGTTTT-3', GAPDH sense 5'-GAGTCAACGGATTTGGTCGT-3', and GAPDH antisense5'-GACAAGCTTCCCGTTCTCAG-3'. PCR was performed usingPusion High-Fidelity DNA polymerase (Thermo Scientific). The cycling conditions were as follows: 35 cycles of 95°C for 10 s, 49°C for 20 s and 72°C for 20s. For quantification, each sample was normalized tohuman GAPDH as a reference.

Protein isolation and western blotting

A subcellular fractionation protocol provided by Thermo Scientific (Cat No.78835;Waltham; MA) was used to isolate cytosolic and nuclear protein from cells. The protein concentrations of the cell lysates were measured using the Pierce BCA Protein Assay Reagent Kit (Cat No. 23225). Proteins were separated by 15% SDS-PAGE. After electrophoresis, proteins were transferred to a polyvinylidenedifluoride membrane (Millipore, Bedford, MA). The membrane was incubated with anti-HMGB1 antibody (Cat No. ab18256;Abcam), anti-p-eIF2α antibody(Cat No. ab32157;Abcam), anti-PERK antibody(Cat No. 5683;Cell Signaling Technology), anti-XBP-1 antibody(Cat No. ab37152;Abcam), anti-lamin A+C antibody (Cat No. ab108595;Abcam)overnight at 4°C, followed by incubation with secondary antibodies for 1 h at room temperature. The Promega Western Blot Detection System (Cat No. W1008;Madison; WI) was used to detect immunoreactive proteins. GelQuant.NET software provided by biochemlabsolutions.com was used for quantification.

SUPPLEMENTARY FIGURES

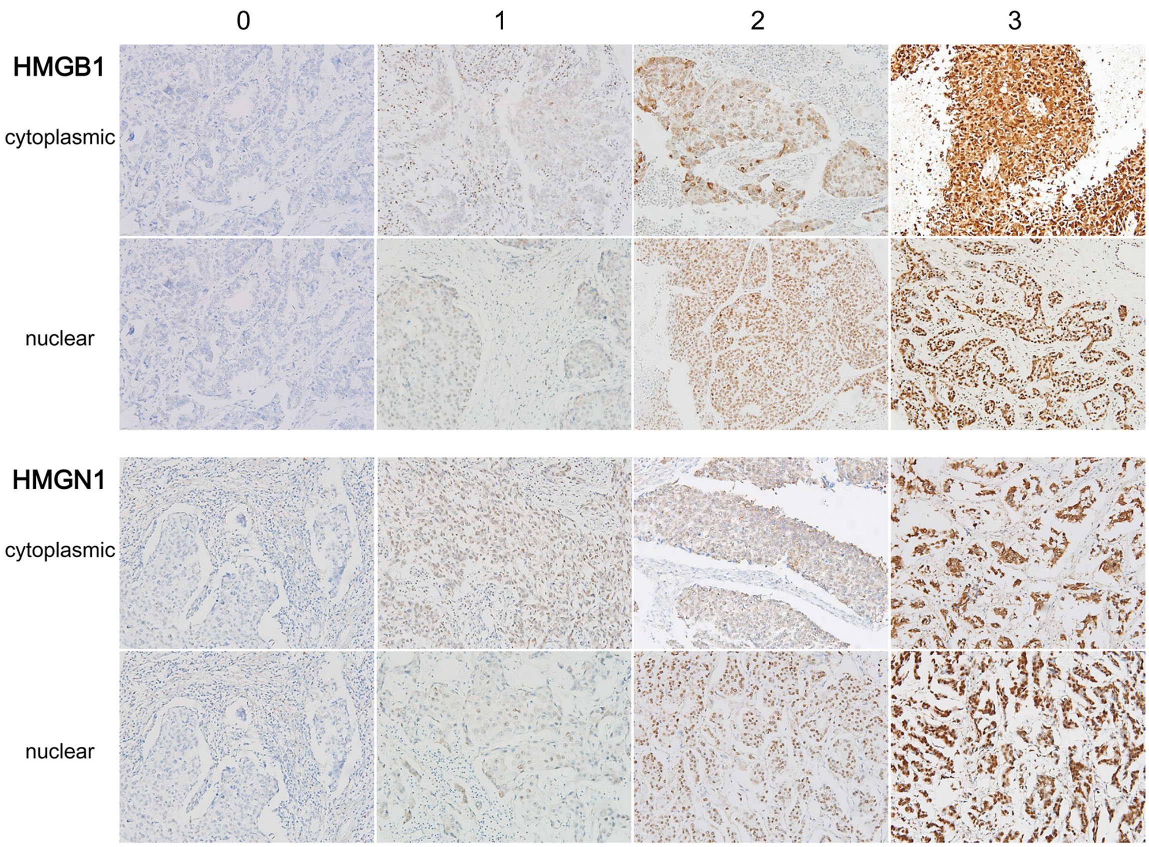

Supplementary Figure S1: Various expression level of HMGB1 and HMGN1 in cytoplasm and nucleus of tumor cells. Each protein expression was evaluated as a four-value intensity score (0; none, 1; weak, 2; moderate, and 3; strong).

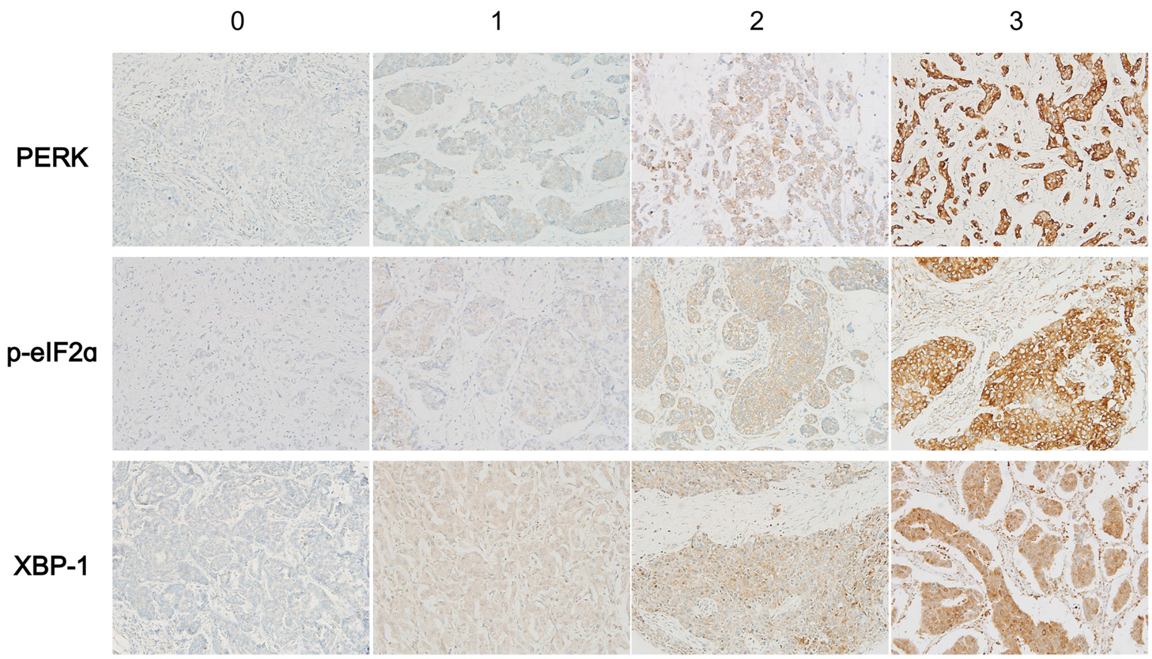

Supplementary Figure S2: Various expression level of the three endoplasmic reticulum-associated molecules in cytoplasm and nucleus of tumor cells. Each protein expression was evaluated as a four-value intensity score (0; none, 1; weak, 2; moderate, and 3; strong).

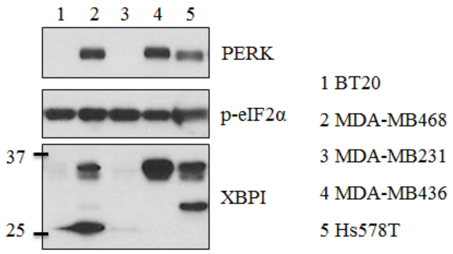

Supplementary Figure S3: The expression of three ERS markers in triple negative breast cancer cell lines. The total protein was extracted from triple negative breast cancer cell lines (BT20, MDA-MB468, MDA-MB231, MDA-MB436 and Hs578t) and seperated on SDS-PAGE gels. Expression of three ERS markers such as PERK, XBPI and p-eIF2a was detected using specific antibobies. XBP-1U and 1S isoform were detected on different size in each cell lines.

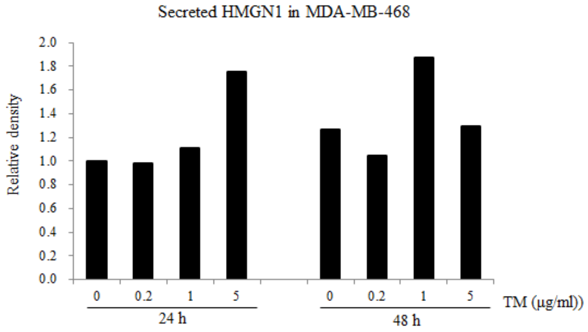

Supplementary Figure S4: Quantification of HMGN1 band density. Secreted level of HMGN1 from MDA-MB-468 upon ERS condition (Figure 1B) was quantified using GelQuant.NET software provided by biochemlabsolutions.com.

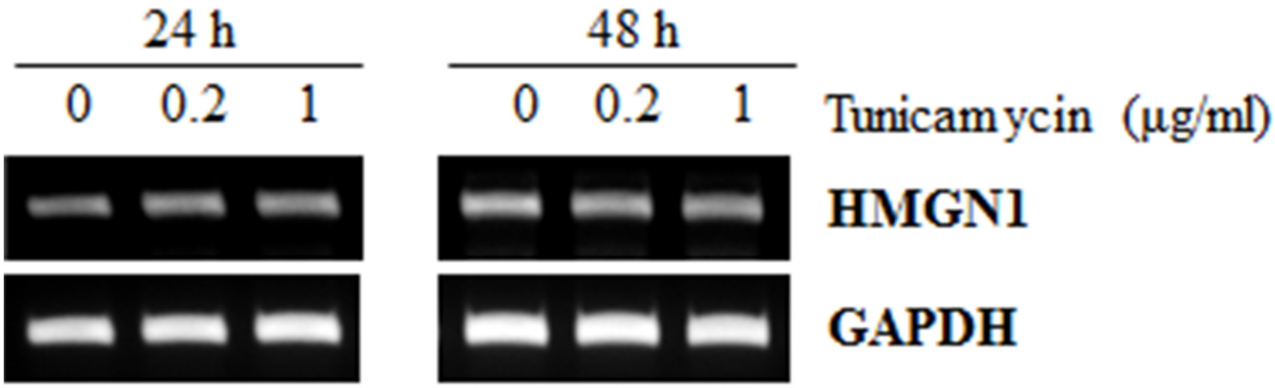

Supplementary Figure S5: mRNA expression of HMGN1 after ERS induction. mRNA expression of HMGN1 was evaluated using HMGN1 specific primers after tunicamycin treatment in MDA-MB-468. GAPDH expression was confirmed as a internal standard.

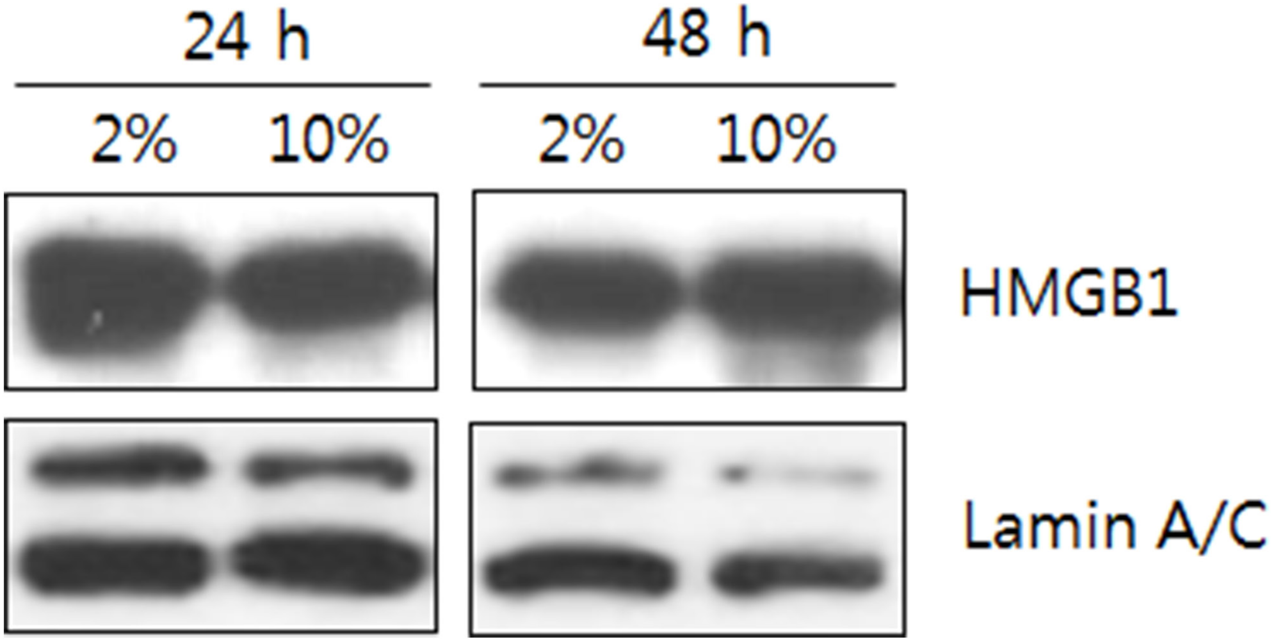

Supplementary Figure S6: Protein expression of HMGB1 in MDA-MB-468. Nuclear protein was extracted from MDA-MB-468 cultured with DMEM containing 2% FBS or 10% FBS. The cells was not fully confluent on harvest time. Nuclear expression of HMGB1 in MDA-MB-468 was decteced. Lamin A/C expression was detected as a positive control.
